# Total knee arthroplasty in patients with haemophilic arthropathy is effective and safe according to the outcomes at a mid-term follow-up

**DOI:** 10.1186/s10195-022-00648-5

**Published:** 2022-07-11

**Authors:** Rui Wang, Zhengming Wang, Yong Gu, Jingjing Zhang, Penghe Wang, Peijian Tong, Shuaijie Lv

**Affiliations:** 1 Zhejiang Chinese Medical University, Hangzhou, Zhejiang China; 2 Shi’s Center of Orthopedics and Traumatology, Shuguang Hospital Affiliated to Shanghai University of Traditional Chinese Medicine, Shanghai, China; 3 Institute of Traumatology & Orthopedics, Shanghai Academy of Traditional Chinese Medicine, Shanghai, China; 4 The First Affiliated Hospital of Zhejiang, Chinese Medical University, 54 Youdian Road, Hangzhou, 310006 Zhejiang Province China

**Keywords:** Haemophilia, Arthropathy, Total knee arthroplasty, Mid-term outcome

## Abstract

**Background:**

Haemophilic arthropathy (HA), a common complication of haemophilia, is secondary to recurrent joint bleeding and increases the prevalence of end-stage osteoarthritis (OA). Total knee arthroplasty (TKA) is a reliable treatment for haemophilia patients. This study was performed to evaluate the mid-term outcomes of TKA for end-stage HA. We hypothesized that the rate of complications of TKA is higher for patients with haemophilia than for patients without haemophilia.

**Methods:**

Patients with HA undergoing TKA from January 2015 to December 2016 in our centre were retrospectively reviewed. All patients were managed by a multidisciplinary team. The improvements in flexion contracture, range of motion (ROM), Knee Society Score (KSS; clinical and functional), Visual Analogue Scale (VAS) score, and satisfaction at final follow-up were analysed to evaluate the effectiveness of TKA in HA. The complications were analysed to evaluate the safety of TKA in HA.

**Results:**

Twenty-eight patients (32 knees) were included in the study. The follow-up was 69.1 ± 5.1 months. Significant differences between the preoperative and final follow-up values of flexion contracture (which changed from 21.1 ± 6.5° to 14.3 ± 4.1°, *P* < 0.001), ROM (from 53.9 ± 15.0° to 70.3 ± 16.3°, *P* < 0.001), clinical KSS (from 33.5 ± 14.4° to 62.7 ± 9.5°, *P* < 0.001), functional KSS (from 46.1 ± 15.5° to 62.9 ± 9.7°, *P* < 0.001), and VAS score (from 6.8 ± 1.4 to 4.9 ± 1.3, *P* < 0.01) were observed. Importantly, the incidence of complications was 15.6% and the satisfaction was 100% in our mid-term study.

**Conclusion:**

Under elaborative and comprehensive management, TKA is effective and safe in patients with advanced HA on the basis of mid-term follow-up outcomes.

## Introduction

Haemophilia is a rare X-linked congenital bleeding disorder characterized by a deficiency in coagulation factors (CFs). It can be divided into two types: haemophilia A and B. The prevalence of haemophilia A is 1/5000, and that of haemophilia B is 1/25000 [1, 2]. It was shown that joint bleeding may occur as frequently as 20–30 times per year in severe hemophilia, which refers to FVIII or FIX activity that is under 1% of the normal activity [3]. Due to the repeated bleeding in the joints, the intra-articular deposition of haemosiderin and iron, and the participation of inflammatory factors, haemophilia is always accompanied by haemophilic arthropathy (HA), which comes with bone destruction, joint deformities, and osteoporosis [4, 5]. For patients with advanced HA, arthroplasty is regarded as the best option [6, 7]. Beneficial outcomes in terms of postoperative pain and function have been reported by some studies [6–8]. However, in some of those studies, patients were followed up for only a short time. It was reported that haemophilia patients have a higher risk of complications such as infection, prosthesis loosening, and postoperative haematoma after arthroplasty [9–11]. The purpose of this article was twofold: (1) to review the functional outcomes and satisfaction of haemophilia patients who underwent total knee arthroplasty (TKA) and (2) to describe the clinical and surgical considerations when managing these patients. Our hypothesis was that the clinical outcomes would be improved after TKA in haemophilia patients, but that there would be several complications, including intra-articular bleeding and earlier aseptic loosening.

## Patients and methods

### Study design

All patients with haemophilia who underwent TKA between January 2015 and December 2016 in our centre were reviewed. All patients had read and signed an informed consent form. Inclusion criteria were end-stage haemophilic arthropathy of the knee with a Modified Arnold–Hilgartner grade of IV, complete destruction of articular cartilage, severe pain, functional impairment, and no response to conservative treatment [12]. Patients who were unable to tolerate TKA, had previous knee surgeries, or had a recent infection in the knee were excluded. All patients were visited at 6 months, 2 years, and 5 years postoperatively.

### Perioperative haematological management and radiographic examination

Preoperatively, standard radiographs (AP view, lateral view, and long-leg standing radiographs) were taken. Knee CT and 3D reconstruction were performed to assess bone defects. As well as routine haematological and biochemical analyses, CF inhibitor and pharmacokinetic tests were performed. Using the World Federation of Hemophilia (WFH) guidelines [13], the CF dosage was adjusted according to the pharmacokinetic test results. During the perioperative period, intravenous bolus injection of the coagulation factor was performed. Based on a combination of the WFH guidelines and our experience, the management procedures in our centre were as follows: pre-infusion of CFs was performed before the operation, and the peak in the CF activity and the attenuation in vivo after infusion were monitored. The dosage and interval of the perioperative medication were formulated based on the results. Usually, the CF activity was supplemented to above 80% during the operation, maintained above 60% within 1–3 days after the surgery, and maintained above 40% for 4–7 days after surgery.

No patient received pharmacologic prophylaxis for thromboprophylaxis. We applied graduated compression stockings (GCS) and an intermittent pneumatic compression device (IPCD) as mechanical prophylactic methods for all patients.

### Surgical technique

A tourniquet was performed on all patients until the skin closure had finished. The arthroplasty procedures were carried out by the same senior surgeons, using a midline anterior incision with a medial parapatellar approach. Isotonic saline solution (0.2 g tranexamic acid (TXA) in 250 ml normal saline) was applied for wound irrigation. Topical TXA (800 mg) was applied intra-articularly to each operated joint after skin closure. For 3–5 days postoperatively, prophylactic antibiotics (xefuroxime 1.5 g) were administered by intravenous infusion twice a day. We did not use suction drains on our patients.

If there is a severe deformity or medial collateral ligament insufficiency in haemophilia, it is necessary to sacrifice the posterior cruciate ligament (PCL), use a condyle-restrictive (LCCK) prosthesis, and to perform an extensive posterolateral soft-tissue release to correct deformity. However, it is necessary to be aware that a large amount of lateral soft-tissue release may cause common peroneal nerve palsy and knee instability. In our opinion, the principle of prosthesis selection is that the degree of restriction of the prosthesis should be as low as possible under the condition of maintaining joint stability. Bone transplantation was employed to fill bone defects using cement and metal spaces, depending on the size of the defect. All cases received a cemented posterior-stabilized (PS) knee prosthesis. All knees received Stryker's prosthesis (Stryker^®^, Stryker Scrorpio NRG Knee-flexed).

### Postoperative rehabilitation

Postoperative rehabilitation started 24 h after the surgery under the guidance of the rehabilitation physician. The rehabilitation protocol included passive and active mobilization of the knee, quadriceps exercises, and proprioceptive, gait, and functional retraining (walking and activities of daily living). Patients who had undergone TKA performed continuous passive motion exercises in the early stage. Passive and active mobilization of the knee and quadriceps exercises were encouraged 1 h after the infusion of coagulation factors (with factor levels maintained at 40–60%) and stopped after 2 h. During rehabilitation, it was of great importance to monitor clotting factor concentrations and joint bleeding. If joint bleeding occurred, rehabilitation was immediately stopped and the RICE (rest, ice, compression, and elevation) method was applied [14].

### Outcome measurement

#### Improvement of knee joint function and pain

The improvement of knee joint function mainly included the correction of flexion deformity, the change in joint range of motion (ROM), and the Knee Society Score (KSS) enhancement. The Visual Analogue Scale (VAS) was applied to evaluate the alleviation of pain.

#### Complications and satisfaction

Complications were recorded, such as the development of CF inhibitors, wound complications, infections, joint bleeding, thrombosis, and prosthesis loosening. Patient satisfaction was assessed, with five possible levels: disappointed, dissatisfied, neutral, satisfied, and very satisfied. Those answers were scored 1–5, respectively, on a Likert scale [15].

### Statistical analysis

All data analyses were performed using SPSS version 25.0 (IBM, Armonk, NY, USA). Mean ± standard deviation or frequency (percentage) was used to summarize variables, as appropriate. The Shapiro–Wilk test was applied to test the normality of data. The differences between preoperative and postoperative values of continuous variables were calculated using Student’s* t*-test or the Wilcoxon Mann–Whitney test. A *p* value of < 0.05 was considered statistically significant.

## Results

### Demographics

Twenty-eight patients (32 knees) were included in the study. All patients were male, with an average age of 38.6 years and a BMI of 22.2 kg/m^2^. Twenty-four patients had haemophilia A and 4 patients had haemophilia B. Among the patients, 8 had mild haemophilia (CF activity 5–40%), 13 had moderate haemophilia (CF activity 1–5%), and 7 had severe haemophilia (CF activity < 1%). Twenty-two patients were infected with the hepatitis C virus (HCV) and 6 patients were infected with the human immunodeficiency virus (HIV). Six patients had both HCV and HIV. The mean follow-up was 69.1 months and the mean length of hospital stay (LOS) was 26.3 days. The patient demographics are shown in Table 1.

**Table 1:** Patient demographics

Variable	
Male (*n*/%)	28/100%
Age (years)	38.6 ± 9.4
BMI (kg/m^2^)	22.2 ± 3.6
Type of haemophilia	
Haemophilia A	24/88.0%
Haemophilia B	4/12.0%
Severity of haemophilia	
Mild	8/28.5%
Moderate	13/46.4%
Severe	7/25.0%
Coinfections	
HCV (*n*/%)	22/79.0%
HIV (*n*/%)	6/21.0%
HCV and HIV (*n*/%)	6/21.0%
LOS (days)	26.3 ± 9.1
Follow-up (months)	69.1 ± 5.1

*BMI* body mass index,* LOS* length of hospital stay,* HCV* hepatitis C virus,* HIV* human immunodeficiency virus

### Improvement of knee joint function and pain

**Fig. 1:**
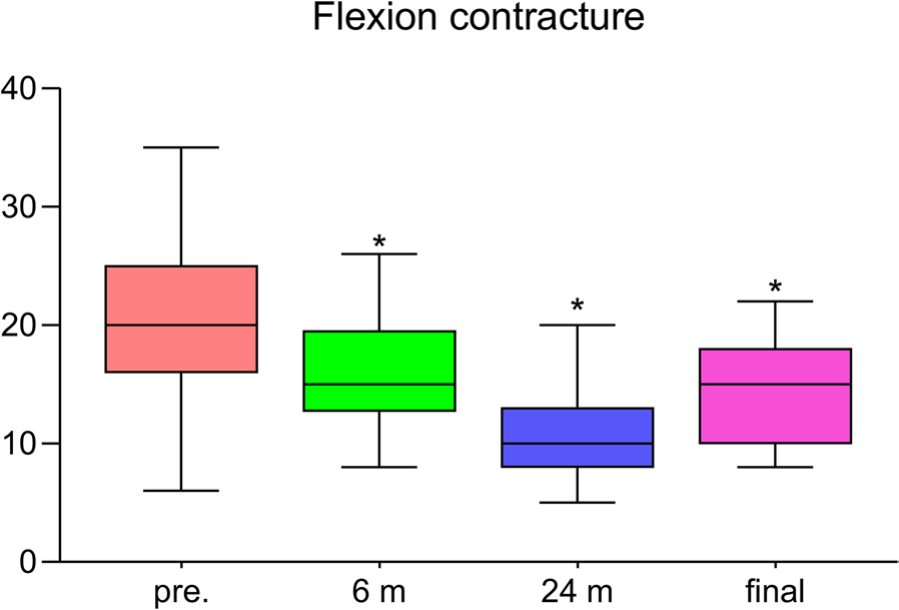
Trend in the flexion contracture, with the results obtained preoperatively, at 6 and 24 months postoperatively, and at the final follow-up shown.* Pre*. preoperative; ^*^ *P* value < 0.05 versus the preoperative value

**Fig. 2:**
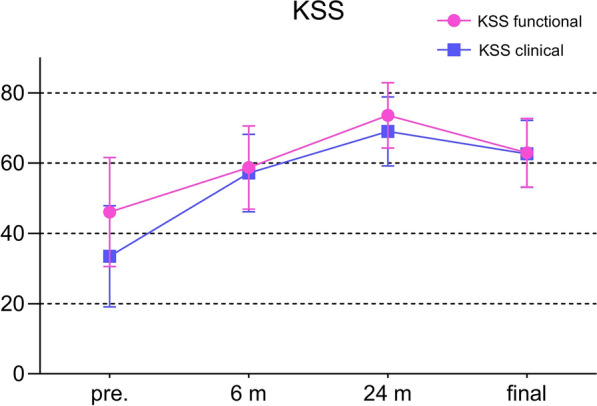
Trends in the clinical KSS and functional KSS, with the results obtained preoperatively, at 6 and 24 months postoperatively, and at the final follow-up shown.* KSS* Knee Society Score,* Pre.* preoperative

**Fig. 3:**
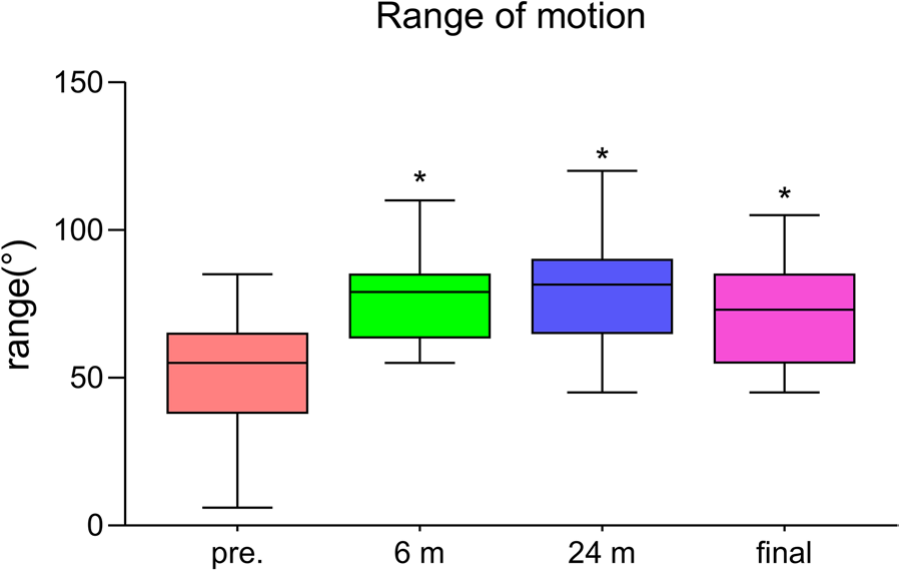
Trend in the ROM, with the results obtained preoperatively, at 6 and 24 months postoperatively, and at the final follow-up shown.* ROM* range of motion,* Pre*. preoperative; ^*^ *P* value < 0.05 versus the preoperative value

**Fig. 4:**
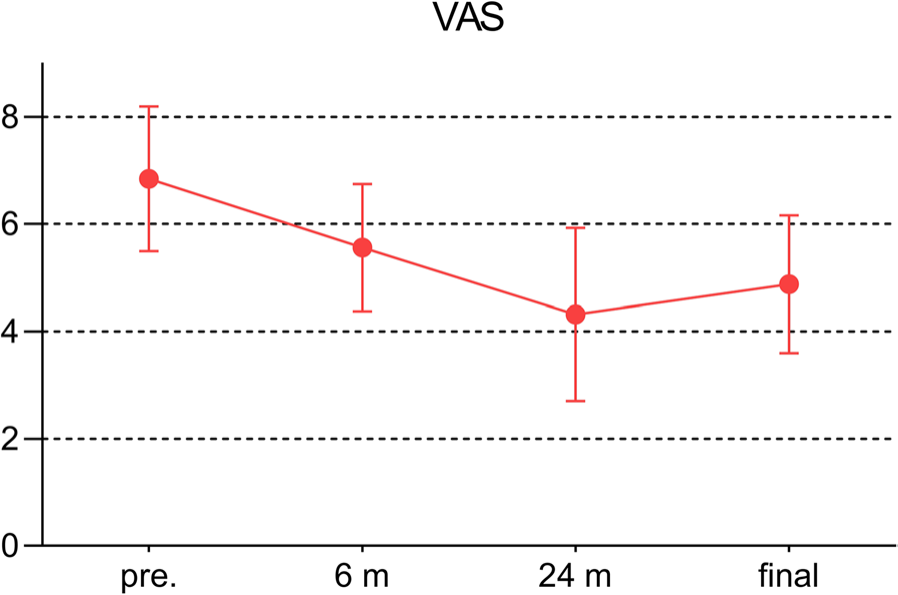
Trend in the VAS score, with the results obtained preoperatively, at 6 and 24 months postoperatively, and at the final follow-up shown.* VAS* visual analogue scale,* Pre*. preoperative

The flexion contracture decreased from 21.1 ± 6.5° to 14.3 ± 4.1° (*P* < 0.001, Fig. 1). Similarly, the clinical KSS and the functional KSS greatly improved, shown in Fig. 2 (*P* < 0.001, respectively). The ROM increased from 53.9 ± 15.0° to 70.3 ± 16.3° (*P* < 0.001, Fig. 3). The VAS score changed from 6.8 ± 1.4 to 4.9 ± 1.3 (*P* < 0.01, Fig. 4). Thus, there were significant differences between the preoperative and final follow-up values of flexion contracture, ROM, clinical KSS, functional KSS, and VAS, as shown in Table 2.

**Table 2:** Comparison of function and pain preoperatively and at the final follow-up

Variable	Preoperative	Final follow-up	*P* value
Flexion contracture (°)	21.1 ± 6.5	14.3 ± 4.1	0.00^*^
ROM (°)	53.9 ± 15.0	70.3 ± 16.3	0.00^*^
Clinical KSS	33.5 ± 14.4	62.7 ± 9.5	0.00^*^
Functional KSS	46.1 ± 15.5	62.9 ± 9.7	0.00^*^
VAS score	6.8 ± 1.4	4.9 ± 1.3	0.00^*^

*KSS* Knee Society Score,* VAS* Visual Analogue Scale,* ROM* range of motion

^*^ *P* value < 0.05 versus preoperative value

### Complications and satisfaction

**Fig. 5:**
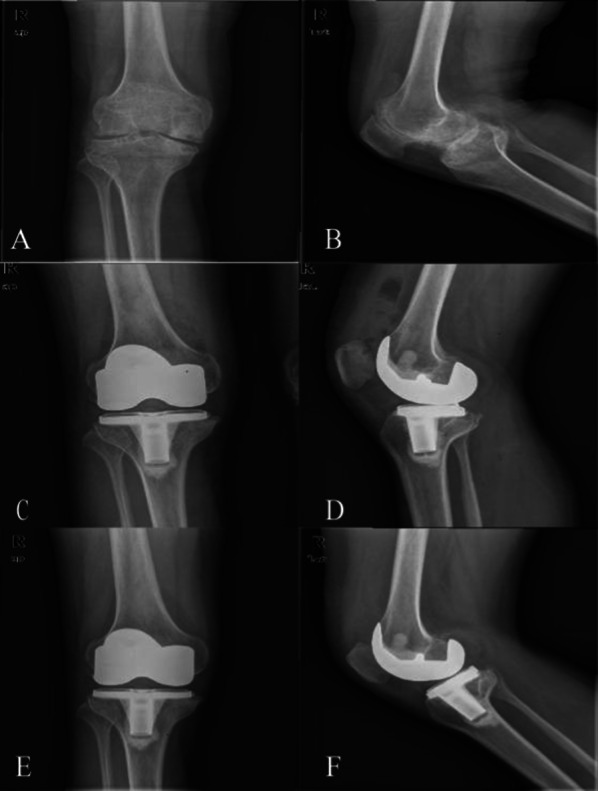
Radiographs of a 35-year-old male patient with severe haemophilic arthropathy. Preoperative X-rays showed extensive destruction of an articular surface, narrowing of the joint space, and a square patella. **A** Preoperative anteroposterior radiograph. **B** Preoperative lateral view. **C** Postoperative anteroposterior radiograph. **D** Postoperative lateral view. **E** Final follow-up anteroposterior radiograph. **F** Final follow-up lateral view

**Fig. 6:**
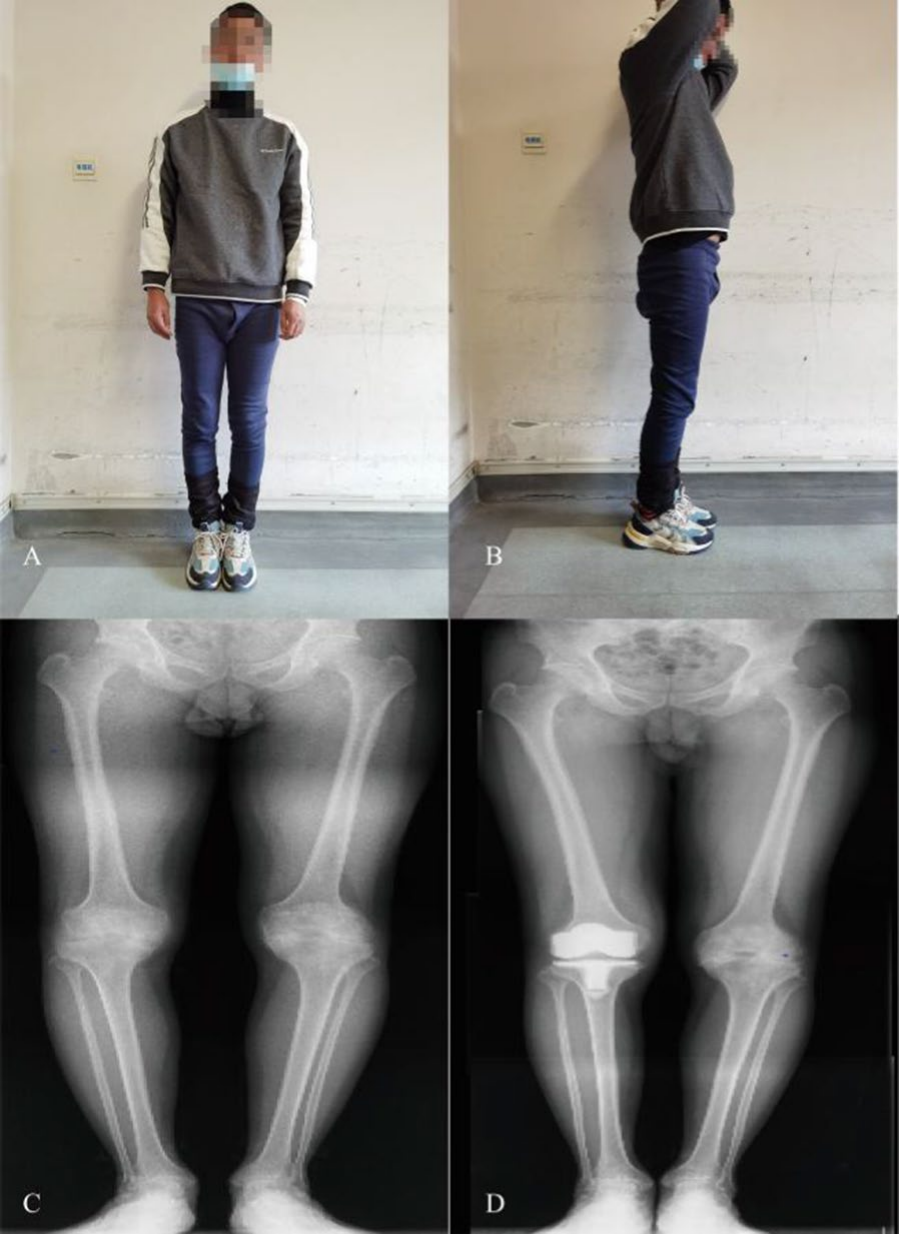
Clinical and radiographic views of both lower extremities. Postoperative X-rays showed a good position of the prosthesis and a good restoration of the force line of the right lower limb

There were no patients with thrombosis and inhibitors. Only one patient with redness and swelling of the postoperative wound during the postoperative recovery period was observed. There were two cases of tension blisters of incision, which were cured after repeated dressing changes. One patient suffered a partial tear of the quadriceps tendon, which was healed by conservative immobilization treatment. A patient was considered to have suspicious prosthesis loosening based on an imaging examination, which was treated with low-intensity knee muscle exercise and by taking anti-osteoporosis drugs. There was no prosthesis loosening at our last follow-up. Surprisingly, the patients were very satisfied with 25 knees and satisfied with 7 knees, meaning that the satisfaction rate was 100%. These results are shown in Table 3. Two typical cases are shown in Figs. 5, 6.

**Table 3:** Complications and satisfaction

Variable	
Complications	15.6%
Redness and swelling of incision	1/3.1%
Tension blisters of incision	2/6.3%
Injury of quadriceps femoris	1/3.1%
Suspicious prosthesis loosening	1/3.1%
Infection	0
DVT	0
Satisfaction	32/100%
Very satisfied	25/78.1%
Satisfied	7/21.9%
Disappointed	0/0%
Dissatisfied	0/0%
Neutral	0/0%

*DVT* deep vein thrombosis

## Discussion

TKA is known as the most suitable orthopaedic treatment for end-stage haemophilic arthropathy. Due to the special features of haemophilia, such as a bleeding tendency, prolonged bony deformity, soft tissue contracture, and muscle atrophies [16], the surgical procedure is more difficult than TKA in knee osteoarthritis. However, due to an improvement in haematological management and a deeper understanding of the pathophysiology of haemophilic arthropathy, it is easier to perform TKA in patients with haemophilic arthropathy.

### Range of motion and satisfaction

When compared with non-haemophilia patients, patients with haemophilic arthropathy of the knee tend to have a poorer postoperative functional outcome. In a study of 20 patients with haemophilia, the mean ROM at last follow-up was 92° and the mean preoperative flexion contracture was 3° [17]. In a cohort of 116 haemophilic procedures at a single institution, a 5° (5° to 0°) improvement in flexion contracture and a 15° (75° to 90°) improvement in ROM were reported [18]. In our cohort, the average ROM of the knee joint at the last follow-up was 70.3° and the average flexion contracture was 14.3°. Both the clinical score and the functional score of the KSS significantly improved from 33.5 preoperatively to 62.7 at the last follow-up (*P* < 0.001) and from 41.6 preoperatively to 62.9 at the last follow-up (*P* < 0.001), respectively. Compared with the above literature, our improvements in maximum postoperative ROM and flexion contracture were not significant. Similar to our study, there was no significant difference between the pre- and postoperative ROM in a study by Westberg et al., with a total ROM of 70° and 79°, respectively [8]. Indeed, Ernstbrunner et al. reported that the total flexion in haemophiliac patients slight decreased postoperatively (from 89° to 87°) [19]. We believe that patient compliance and severe soft-tissue contracture are the two main reasons for the poor postoperative ROM in our cohort. Although our cases showed a 7° improvement in flexion contracture postoperatively, it was still relatively high, which hindered the postoperative rehabilitation process and led to a poor ROM. Also, low patient compliance with postoperative rehabilitation resulted in a poor ROM and high flexion contracture. In this series, the average postoperative VAS score was 4.9, which is connected to irregular prophylaxis with CFs. The average Knee Society Score was also less than in the previous literature [20–22]. These results might be associated with the abovementioned restricted ROM, VAS score, and flexion contracture postoperatively.

Surprisingly, 100% of our patients were satisfied with the procedure. In a systematic review, Naoki Nakano et al. [23] concluded that a negative history of mental health problems, severe preoperative radiological degenerative change, no/less postoperative pain, good postoperative physical function, and preoperative expectations being met contributed to better patient satisfaction following a TKA. A study by Noble et al. showed that patients’ expectations were highly correlated with their satisfaction after total knee replacement [24]. With their serious functional impairment and extreme pain preoperatively, the daily needs of our patients were unfulfilled before TKA. For activities of daily living, 65–70° of knee flexion are necessary for the swing phase of normal gait, whereas at least 90° are needed to descend stairs and 105° to rise from a low chair [25]. In our cases, the VAS score decreased from 6.8 to 4.9 and maximum ROM increased from 53.9° to 70.3°. The low expectations of our patients, their strong relief from pain, and their fulfillment of daily activities were considered to be important factors leading to the excellent patient satisfaction at our centre. Besides, our centre had a matching policy to reduce the hospital fees for haemophilia. 

### Main complications

Interestingly, our study showed that the incidence of complications was 15.6%, which is lower than reported in the literature [26, 27]. We think that this difference is associated with our own unique insights into surgical techniques, infection management, and rehabilitation. Infection, prosthesis loosening, periprosthetic fractures, prosthesis removal, bleeding, neurovascular injury, and inhibitor development are some common problems that occur after TKA in haemophilia patients. We now discuss the main complications that arose in our study.

#### Knee extensor tendon injury

It was reported that the incidence of knee extensor tendon injury was approximately 1–12% after TKA. Because of the marked adhesions, arthrofibrosis, and decreased ROM in haemophilia patients, patellar subluxation is restricted, making exposure difficult. We usually used a modified quadriceps snip technique that we call Z-shaped quadricepsplasty to resolve knee intra-articular adhesions. Firstly, two small vertical incisions were made on both sides of the rectus femoris muscle. Then, the rectus femoris was divided into two parts after the Z-myotomy. Finally, the two parts of the cut muscle were sutured together. There was a suspected case of partial quadriceps tendon rupture among our follow-up cases. Here are some possible reasons. On the one hand, an incision of the rectus femoris was performed, which could destroy the blood supply to the tendon and easily cause damage to the quadriceps tendon. On the other hand, long-term muscle atrophy, recurrent bleeding, and unfavorable muscle conditions are potential risk factors. Besides, the premature time and the improper intensity of postoperative rehabilitation were harmful to knee extensor tendon.

#### Prosthesis aseptic loosening

Prosthesis aseptic loosening is a key complication that is worthy of attention, especially in people with haemophilia. The incidence of aseptic loosening after the first TKA is 1–23% for ordinary people without haemophilia [28, 29]. The young age, poor bone quality, microhaemorrhages, and prevalence of osteoporosis [30] in patients with haemophilia are strongly associated with prosthesis loosening. Song et al. [11] reported that there was no wear or loosening after TKA in a study with a mean follow-up of 6.8 years. In our study, a patient's intraoperative and postoperative imaging examinations showed possible prosthesis loosening. Daily low-intensity exercise, anti-osteoporosis drugs, and CF replacement therapy were recommended for patients. When reasonable preventive measures were applied, there was no occurrence of early prosthesis loosening until the last follow-up in this patient.

#### Infection

Furthermore, infection, an unavoidable topic, was historically considered a catastrophic barrier in haemophilia patients [31]. It was recognized that haemophilia was accompanied by multiple blood-borne viral infections, such as HIV and hepatitis virus, which affects patient immunity and raises the risk of infection [32, 33]. However, some recent studies [34, 35] of TKA in patients with HA have shown that the postoperative infection rate is very low. Our results are consistent with theirs, which indicates that infection is no longer a major obstacle to elaborative and comprehensive management.

#### Deep vein thrombosis

Deep venous thrombosis (DVT) is one of the most common complications after artificial joint replacement. The use of a tourniquet, immobilization, long-term bed rest, endothelial vascular injury, trauma, and hypercoagulability [36, 37] are associated with DVT. Buckner et al. [38] reported that the incidence of symptomatic venous thromboembolism (VTE; 4.3%) was similar to its estimated incidence in the general population without thromboprophylaxis. A study [39] in Japan did not detect any DVT. Similiarly, there was no DVT in our patients who had undergone TKA. Opinions in the literature on the need for VTE prophylaxis in patients with haemophilia differ [40, 41]. Due to their lack of congenital CFs, haemophilia patients have a low risk of DVT. Even though haemophilia patients were given CFs during surgery, they did not reach a hypercoagulable state. We believe that there is no need for routine pharmacologic prophylaxis after the operation, but mechanical prophylaxis should be employed, such as graduated compression stockings, intermittent pneumatic compression devices, and venous foot pumps.

Undoubtedly, there are some limitations to our study. The principal limitation is the retrospective nature of the work, with no control group. Additionally, the number of patients is relatively small. A large-sample-size study should be performed. Also, this is a single-centre study, so its results may not apply to all centres. However, the follow-up in our study is relatively long compared to those of other studies reported in the literature, and it adds to the available knowledge on the results of TKA in haemophilia patients.

## Conclusion

TKA can improve joint function and relieve pain in patients with HA. It is the best choice for end-stage HA.

## Data Availability

The datasets used and analysed during the current study are available from the corresponding author on reasonable request.

## Abbreviations

HA: Haemophilic arthropathy
OA: Osteoarthritis
ROM: Range of motion
KSS: Knee Society Score
VAS: Visual Analogue Scale
CFs: Coagulation factors
TKA: Total knee arthroplasty
WFH: The World Federation of Hemophilia
CPM: Continuous passive motion
HCV: Hepatitis C virus
HIV: Human immunodeficiency virus
LOS: Length of hospital stay
PCL: Posterior cruciate ligament
BMI: Body mass index
DVT: Deep vein thrombosis
VTE: Venous thromboembolism
GCS: Graduated compression stockings
IPCD: Intermittent pneumatic compression device
